# Physiotherapy management of first nations children with bronchiectasis from remote top end communities of the northern territory: a retrospective chart audit

**DOI:** 10.3389/fped.2023.1230474

**Published:** 2023-10-13

**Authors:** A Welford, GB McCallum, M Hodson, H Johnston

**Affiliations:** ^1^ Community Allied Health Team, Top End Population and Primary Healthcare, NT Health, Darwin, NT, Australia; ^2^ Child Health Division, Menzies School of Health Research, Charles Darwin University, Darwin, NT, Australia

**Keywords:** bronchiectasis, physiotherapy, management, children, first nations, remote communities, guidelines

## Abstract

**Background:**

Bronchiectasis is a chronic pulmonary disorder which is prevalent among Australian First Nations people in the Northern Territory (NT). Current guidelines recommend physiotherapy as part of multi-disciplinary management of children with bronchiectasis, however in our setting, involvement of physiotherapy remains unknown. We thus undertook a retrospective chart audit to examine physiotherapy management of First Nations children (<18 years) from remote First Nations communities in the Top End of the NT at the index bronchiectasis diagnosis and 12 months following diagnosis.

**Methods:**

Participants were identified from a larger prospective study of children investigated for bronchiectasis at Royal Darwin Hospital, NT (2007–2016). Children were included if they were First Nations, aged <18 years, had a radiological diagnosis of bronchiectasis on high resolution computed tomography scan and lived in a remote community serviced by NT Government health clinics. The medical records from NT Government hospitals, health clinics and where possible other medical service attendance were reviewed for physiotherapy referral and management at the time of bronchiectasis diagnosis and in the following 12 months in the community.

**Results:**

Of 143 children included, the mean age was 3.1 (standard deviation 2.4) years and 84 (58.7%) were males. At the index diagnosis, 76/122 (62.3%) children were reviewed by a physiotherapist, consisting of airway clearance techniques (83.8%), physical activity/exercise (81.7%) and caregiver education (83.3%), with only 7/127 (5.5%) having evidence of referral for community-based physiotherapy. In the following 12 months, only 11/143 (7.7%) children were reviewed by a physiotherapist, consisting of airway clearance techniques (54.5%), physical activity/exercise (45.5%) and caregiver education (36.4%).

**Conclusion:**

This study demonstrates a significant gap in the provision of physiotherapy services in our setting and the need to develop a standardized pathway, to support the best practice management of children with bronchiectasis in remote Top End communities of the NT.

## 1. Introduction

### 1.1 Best practice physiotherapy management for childhood bronchiectasis

Once considered rare, the profile of bronchiectasis in respiratory medicine has gained importance over several decades and has been recently termed “an emerging global epidemic” (1). Although published data remains limited in childhood, bronchiectasis is now recognised to exist in all settings globally (2–4) and is estimated to range from 0.2 to 735 per 100,000 children (3, 5), with a higher disease burden and severity reported among First Nations children living in socially disadvantaged populations of high-income countries (6) and those from low-and middle income countries (5, 7). Among the paediatric population, it is increasingly recognized that the progress of bronchiectasis can be halted and/or possibly reversed if treatment is early and optimized (3, 8). This is particularly important in our setting where we have the highest published rates of paediatric bronchiectasis globally (1 in 68 children from Central Australia, Northern Territory) (6). Data from the same region but in First Nations adults further emphasizes this priority, whereby suboptimal management resulted in a >20 year mortality gap, more frequent exacerbations and reduced lung function compared to non-First Nations adults with bronchiectasis (9). Furthermore, a study recently published in our setting demonstrated that while there were some improvements in the clinical profiles of children over two five-year time periods, bronchiectasis remained very high among First Nations children (10). Thus early and optimal management of bronchiectasis in children to prevent airway damage, support lung growth, optimize quality of life and minimize exacerbations across the life course is very important (11, 12).

Several national and international guidelines have been published to inform best practice management of bronchiectasis across the life course, including the role physiotherapy plays in management, such as airway clearance techniques (ACTs), exercise/physical activity and education for parents and caregivers (11, 13–15). ACTs use different breathing patterns, which may be supported by the use of a device, to assist sputum clearance, improve ventilation, and reduce cough frequency (16). For children with bronchiectasis, ACTs should be individualized, developmentally appropriate and taught by a paediatric-trained respiratory physiotherapist (17). Guidelines also recommend that ACTs are reviewed biannually by a physiotherapist, adapted as children mature and are provided more frequently during acute respiratory exacerbations (11). A small number of randomized controlled trials in children and adults with bronchiectasis have reported improved lung function in those receiving ACTs versus control groups (18–20), yet there remains a lack of high-quality evidence supporting their use. This is a significant evidence gap and there have been calls for physiotherapy clinical trials among bronchiectasis patients (21).

Guidelines also recommend regular physical activity for children with bronchiectasis, as it is thought that therapeutic exercise may reduce frequency and severity of acute exacerbations, improve aerobic fitness, improve health related quality of life, and prevent the development of other chronic conditions in children with bronchiectasis (22), yet, there currently remains a lack of evidence in this population (23). The importance of caregiver education is also highlighted in current guidelines, with a role for all multidisciplinary team members to provide education about bronchiectasis, promote individualized and self-initiated management plans and encourage regular airway clearance techniques (11, 13).

It is well documented that First Nations children particularly in the Northern Territory, Australia are disproportionately impacted by bronchiectasis and experience a range of barriers to timely diagnosis and best practice management (12, 24). While guidelines recommend physiotherapy as part of multidisciplinary management of bronchiectasis, access to physiotherapy for First Nations children from remote communities in our setting is currently unknown. Thus, we undertook a retrospective chart review to examine physiotherapy management of First Nations children with bronchiectasis from Top End remote communities of the Northern Territory, immediately following diagnosis of bronchiectasis on high resolution computed tomography scan (HRCT) at Royal Darwin Hospital and in the community 12 months post-diagnosis.

## 2. Methods

### 2.1. Study participants and setting

Study participants were identified from a larger prospective study conducted at Royal Darwin Hospital, Northern Territory, Australia. This prospective study commenced in 2007 and enrolled children aged <18 years undergoing bronchoscopy and/or HRCT for clinical investigation of suspected bronchiectasis (25). Children were included in this current study if they (i) had a radiological diagnosis of bronchiectasis on HRCT in the period from November 2007 to December 2016, (ii) resided in a Top End remote First Nations community; and (iii) accessed services from a Northern Territory Government primary healthcare facility. Ethics approval for this study was received from the Human Research Ethics Committee of the Northern Territory Department of Health and Menzies School of Health Research (HREC 2021-4078) who provided a waiver of consent for this current analysis, as primary caregivers from the original study had provided written consent (HREC 07/63).

### 2.2. Data collection

Demographic, medical history and the index bronchiectasis details were extracted from the original study database and entered into a password protected REDCap database. The medical records were then reviewed to firstly describe inpatient management at the index bronchiectasis diagnosis for evidence of physiotherapy assessment, treatment, education, home exercise programs and referral to community physiotherapy. For outpatient physiotherapy management, the electronic medical records of Northern Territory Government serviced primary healthcare centres were reviewed using the Primary Care Information System and where possible other medical service attendance including assessment, treatment, education, home exercise programs physiotherapy referral, and presentations for community-treated respiratory exacerbations using our established methods (10).

### 2.3. Analysis

Summary statistics are presented as mean and standard deviation (SD), median and interquartile range (IQR: 25%–75%) and frequency (percentage) for categorical data. Data from the original bronchiectasis study was imported into REDCap and data for this current study entered directly into REDCap (26). We analysed the data using Stata version 17.

## 3. Results

### 3.1. Baseline participant characteristics

A total of 143 children were identified for inclusion in this study. The mean age at time of diagnosis was 3.1 (SD 2.4) years and 84 (58.7%) were male. Further baseline characteristics are described in Table 1.

**Table 1:** Baseline demographics, medical history and primary caregiver reported symptoms at index bronchiectasis diagnosis.

Variable	*n* = 143 (%)
Demographics	
Age (years), mean (SD)*	3.1 (2.4)
Male sex	84 (58.7)
Low birth weight, <2.5 kg	51/137 (37.2)
Preterm, <37 weeks	50/138 (36.2)
Immunizations up to date (≥2 doses of pneumococcal vaccines)	140 (97.9)
Respiratory hospitalizations	
Number of hospitalizations (ever) (median, IQR)	2 (1–3)
Number of hospitalizations (last 12 months) (median, IQR)	1 (0–2)
Etiology	
Post infection	140 (97.9)
Chronic neonatal lung disease	3 (2.1)
Primary caregiver reported symptoms	
Cough	
Current cough	87 (60.8)
Chronic cough (daily >4 weeks)	70/133 (52.6)
Wheeze	
Current wheeze	77/137 (56.2)
Previous wheeze	91/126 (72.2)

SD, standard deviation.

Data are presented as number (%) or.

* mean (standard deviation), unless otherwise indicated.

### 3.2. Inpatient physiotherapy management at index diagnosis

Overall, only 122 (85.3%) participant medical records were available in their entirety to review despite multiple attempts to access paper-based copies. Of these, a total of 76 participants (62.3%) had documented evidence of physiotherapy interventions during their index bronchiectasis admission. The types of physiotherapy interventions used are described in Table 2 and largely consisted of airway clearance techniques (83.8%), physical activity/exercise (81.7%), education for primary caregivers (83.3%) and home exercise programs (43.8%). On hospital discharge, referral to outpatient/community physiotherapy occurred for only 7/127 (5.5%) of participants. Three were referred to the outpatient physiotherapy department at Royal Darwin Hospital and four to outreach physiotherapy services (Table 2).

**Table 2:** Physiotherapy review during index hospitalization for bronchiectasis.

Variable	*n* = 143 (%)*
Evidence of physiotherapy review	76/122 (62.3)
Physiotherapy intervention provided	
Airway clearance techniques performed?	62/74 (83.8)
Physical activity/exercise intervention used?	58/71 (81.7)
Education given to primary caregiver?	60/72 (83.3)
Home program provided to primary caregiver?	32/73 (43.8)
Type of home program provided	
Exercise/physical activity	10/73 (13.7)
Airway clearance techniques	22/73 (30.1)
Not specified	41/73 (56.2)
Discharge plan	
Referral to outpatient/community physiotherapy	7/127 (5.5)
What organization child was referred to	
Northern Territory Government outreach physiotherapy	2/7 (28.6)
Royal Darwin Hospital physiotherapy outpatient department	3/7 (42.9)
Private Provider	2/7 (28.6)

* We were unable to physically locate the medical records for 21 children. The electronic discharge summaries at the index bronchiectasis diagnosis admission were also reviewed to identify as much data as possible. The denominator for each question includes only available data and not for the entire 143 children.

### 3.3. Community physiotherapy management 12 months post-diagnosis

In the 12 months following diagnosis, 60/143 (42.0%) participants had documented evidence of a bronchiectasis care plan in their electronic primary healthcare records, with physiotherapy referral only recorded for two children. Overall, only 11 physiotherapy reviews were conducted from 8 participants over the 12 month period (Table 3) which consisted of airway clearance techniques (54.5%), physical activity/exercise (45.5%), education for caregivers (36.4%) and home exercise programs (27.3%). There were 66 separate community-treated respiratory exacerbations over the 12 months, with only two being referred to a physiotherapist for review.

**Table 3:** Physiotherapy management of bronchiectasis in the community 12 months post-diagnosis .

Variable	*n* = 143 (%)
Bronchiectasis care plan and physiotherapy referral	
Evidence of bronchiectasis care plan in the child’s electronic medical records?	60 (42.0)
Evidence of physiotherapist referral for bronchiectasis review within 12 months?	2 (1.4)
Actual number of physiotherapist reviews for bronchiectasis within 12 months	11 (7.7)
Physiotherapy intervention provided	*n* = 11*
Airway clearance techniques performed?	6 (54.5)
Physical activity/exercise intervention used?	5 (45.5)
Education given to primary caregiver?	4 (36.4)
Home program provided to primary caregiver?	3 (27.3)
Community-treated respiratory exacerbations	
Median number of respiratory exacerbations within 12 months post-diagnosis (IQR)	2 (1–3)

IQR, interquartile range.

* A child could have more than one measurement done at a time.

## 4. Discussion

In this study involving 143 children with bronchiectasis from First Nations communities of the Top End of the Northern Territory, we found that at the index diagnosis, most participants (62.3%) were reviewed by a physiotherapist and received interventions in line with current guidelines, including ACTs, exercise, and primary caregiver education. Community-based physiotherapy management was however poor in the following 12 months, with only 7.7% reviewed by a physiotherapist during this time. Our study contributes to the limited data on physiotherapy management in children with bronchiectasis in our setting, by documenting for the first time physiotherapy management at the index diagnosis at Royal Darwin Hospital, and 12 months following diagnosis in remote community settings.

In our study, ACTs were the most frequent intervention provided at bronchiectasis diagnosis and in the following 12 months (83.8% and 54.5% respectfully). This is in line with current recommendations (11), however, there remains no international consensus on what optimal airway clearance technique/s are for children with bronchiectasis with a range of methods described in the literature, including gravity assisted drainage, percussion and vibrations, active cycle of breathing technique, positive expiratory pressure and autogenic drainage (17). Furthermore, guidelines describe a number of factors to consider when selecting ACTs for children, including age, level of maturity and cooperation, clinical presentation and family capacity (17). It is widely acknowledged though that the evidence for ACTs in bronchiectasis is lacking (11, 13, 17, 27), with recent guidelines making strong recommendations for ACTs based on clinical practice and expert opinion, rather than high quality evidence (21). Furthermore, this has been highlighted by parents/caregivers and clinicians as a priority area for bronchiectasis, with calls for large scale clinical trials to determine the utility and efficacy of various ACTs (28).

In other chronic respiratory conditions, it is well documented that exercise training improves cardiovascular fitness and quality of life (29). However, for children with bronchiectasis, physical activity and exercise interventions are poorly documented in the literature and not supported by a strong evidence base, despite being recommended in current guidelines (11). Nonetheless, a small randomized controlled trial from Australia recently observed preliminary evidence for the efficacy of therapeutic exercise in children with bronchiectasis (30). These data are encouraging as the variable and poorly documented exercise interventions provided at diagnosis and in the community in our setting reflect the need for further investment of clinical trials and guidance for physiotherapists around appropriate exercise for children with bronchiectasis.

Similarly, education was provided by most physiotherapists to the caregivers at the index diagnosis (83.3%) and by a smaller number in the community (36.4%). Current guidelines promote education by a multidisciplinary team about recognising exacerbations, developing and initiating self-management plans, minimising exposure to second hand smoke (13), and working alongside interpreters and local health-workers for First Nations people and communities (31). Importantly, at Royal Darwin Hospital, 60%–90% of patients are Aboriginal, and 60% speak an Aboriginal language (32). Despite this, Aboriginal interpreters remain profoundly underutilized (33) and patients frequently report misunderstandings and culturally unsafe communication (34). It is widely documented that providing information is not necessarily effective education and that the quality of communication is key to effective health communication and patient engagement in their healthcare (35). Recent studies from the Northern Territory have described strategies to improve knowledge on common childhood respiratory conditions for First Nations caregivers that include culturally appropriate pictorial-based flipcharts (36) and a multi-lingual app with voiceover in seven First Nations languages and English (37). Physiotherapists and other members of the multidisciplinary team need to work in partnership with children, primary caregivers, families, Aboriginal interpreters, and health services to embed culturally safe communication and education in the management of childhood bronchiectasis. In the Northern Territory, evidence-based guidelines for rural and remote primary healthcare have recently been updated and include a section on paediatric CSLD/bronchiectasis (38). These guidelines highlight the role of all members of the primary healthcare team in supporting best practice management of paediatric CSLD/bronchiectasis, including promoting exercise and airway clearance.

Overall, our study identified a significant disparity between physiotherapy review at the index bronchiectasis diagnosis (62.3%) and in the following 12 months in community (7.7%). For children from remote Top End communities of the Northern Territory, investigation of suspected bronchiectasis requires a tertiary hospital admission for bronchoscopy and/or HRCT (25). This provides a window of opportunity for physiotherapy intervention and accounts for the high proportion of participants seen by a physiotherapist at the time of diagnosis. However, the low referral rate from the tertiary setting to community physiotherapy (5.5%) represents a breakdown in the management pathway for children with bronchiectasis. Guidelines recommend that care plans are developed involving linkages between primary healthcare and hospital facilities (13). In our study, the low referral rate contributed to very few participants receiving physiotherapy follow up in the 12 months following diagnosis. This is inconsistent with current guidelines, which recommend that children with bronchiectasis are reviewed at least biannually by pediatric trained respiratory physiotherapists (11, 13).

Our study also highlights the need to significantly improve referral pathways to physiotherapy in community-based care. Guidelines recommend that ACTs are reviewed and provided more frequently during acute respiratory exacerbations for children with bronchiectasis (11, 13). In the 12 months following diagnosis, 66 separate community-treated respiratory exacerbations were documented which resulted in only two physiotherapy referrals. This represents multiple, missed opportunities for referral to community-physiotherapy and an urgent need to strengthen this care pathway. Furthermore, the high turnover of remote primary healthcare staff of 148% nursing staff and 80% Aboriginal health practitioners per annum, creates significant challenges around interdisciplinary collaboration and optimal care (39).

Access to pediatric trained physiotherapists has been also identified as a barrier by parents of children with bronchiectasis (11, 28). There is well documented undersupply and maldistribution of allied health professionals across rural and remote Australia, substantially increasing with remoteness (40). Access challenges are further compounded by geographical isolation, low population density, poor infrastructure, and high costs of delivering care (41). In our study, we hypothesize that low referral numbers to community physiotherapy were associated with a lack of available services, which was well known to the hospital-based multidisciplinary team. In both acute and community settings, bronchiectasis is chronically underfunded and there is a well-documented inequity in care between bronchiectasis and other respiratory disorders (42). Furthermore, in the Northern Territory, there is no dedicated respiratory physiotherapy service despite the significant burden of disease associated with chronic respiratory diseases such as broncheictasis (25). This study highlights the urgent need to improve access to physiotherapy for children with bronchiectasis in remote Top End communities of the Northern Territory. It also adds further evidence around the need to strengthen comprehensive primary healthcare in remote communities.

The strength of our study is that Royal Darwin Hospital is the sole hospital in the Top End of the Northern Territory that undertakes investigations for bronchiectasis and that we have been prospectively collecting data since 2007, representing one of the largest bronchiectasis cohorts currently available (25). Our study has several limitations that include (1), the retrospective nature, (2) inability to access all medical records in the hospital, (3) not being able to review children’s medical records outside of Northern Territory Government health clinics; and (4) inconsistencies in documentation of physiotherapists in remote communities.

## 5. Conclusion

This study demonstrates a significant gap in the provision of physiotherapy services in our setting and the need to develop a standardized pathway, to support best practice management of children with bronchiectasis in remote Top End communities of the Northern Territory.

## Data Availability

The datasets presented in this article are not readily available because as per our institutions' policies involving Australian First Nations children and in accordance with national guidelines, we are unable to share individual participant data as specific consent for this was not obtained. Requests to access the datasets should be directed to the corresponding author.
